# Lineage‐Biased Neural Stem Cell Grafting Promotes Neuronal Differentiation and Vascular Repair in the Chronic Phase of Stroke

**DOI:** 10.1002/cns.70701

**Published:** 2026-01-04

**Authors:** Tingting Zhang, Da Li, Qibiao Guan, Bin An, Yun Sun, Qiang Wang, Yukai Wang, Baoyang Hu

**Affiliations:** ^1^ State Key Laboratory of Organ Regeneration and Reconstruction, Institute of Zoology, Chinese Academy of Sciences Beijing China; ^2^ Beijing Institute for Stem Cell and Regenerative Medicine Beijing China; ^3^ University of Chinese Academy of Sciences Beijing China; ^4^ National Stem Cell Resource Center, Chinese Academy of Sciences Beijing China

**Keywords:** angiogenesis, cell replacement, cell therapy, chronic phase of ischemic stroke, embryonic body, neural stem cells, neurogenesis, vasculature development

## Abstract

**Aims:**

Ischemic stroke (IS) is a harmful neurological disorder, yet current therapies fail to achieve effective functional neural and vascular restoration. Neural stem cell (NSC) transplantation offers high potential for neuronal replenishment and vascular reconstruction. However, its clinical application is limited by inconsistent in vivo cell fate, unclear therapeutic mechanisms, and variability in differentiation protocols. To address these limitations, we aimed to identify a neural lineage‐biased NSC suitable for neuronal and vascular recovery after IS.

**Methods:**

We assessed two strategies for deriving transplantable NSCs: one involving embryoid body (EB) formation, and the other employing a direct differentiation protocol bypassing EB formation. We compared the cell fate of NSCs derived via both protocols in vitro and following transplantation into mice subjected to IS induced by transient middle cerebral artery occlusion (tMCAO). RNA sequencing and immunofluorescence were used to assess cell fate. Cerebral blood flow (CBF) and behavioral tests were conducted to evaluate functional recovery.

**Results:**

Comparative analyses demonstrated that nEB‐NSCs exhibit elevated neural stemness marker expression, improved neuronal differentiation with reduced astrocytic commitment, and accelerated neurovascular repair kinetics, contributing to considerable motor recovery.

**Conclusions:**

nEB‐NSCs represent a promising cell source for enhancing neurovascular repair and functional recovery following IS.

## Graphical abstract

Schematic diagram comparing two differentiation strategies to generate NSCs. The nEB‐NSCs exhibited a pronounced bias toward neuronal lineage differentiation. Transplantation of nEB‐NSCs into stroke mice (induced by tMCAO surgery) promoted motor recovery and vascular repair.

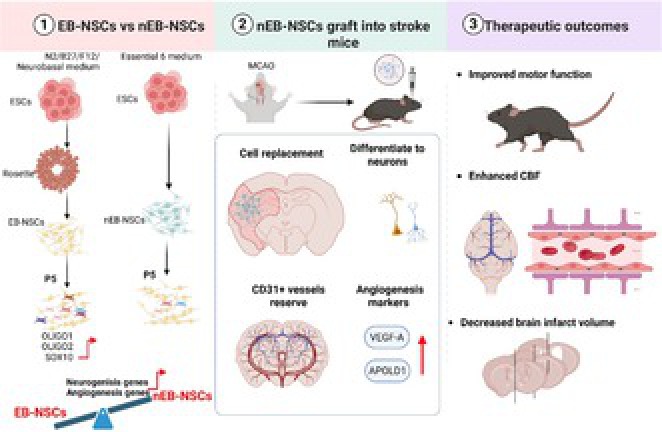

## 1 Introduction

Accounting for approximately 85% of all stroke cases, ischemic stroke (IS) is a severe cerebrovascular disorder that leads to widespread neuronal and vascular endothelial cell death, disrupts neural circuitry, and compromises blood–brain barrier integrity [1]. During cerebral ischemia, the lack of oxygen and nutrient delivery leads to the accumulation of reactive oxygen species, resulting in neuronal death, brain–blood‐barrier disruption, and neuroinflammation [2, 3]. As the condition progresses into the chronic phase, ongoing neuroinflammation and the formation of glial scars impede neuronal regeneration and neural circuit reconstruction, ultimately making motor function recovery more challenging [4, 5].

NSCs can differentiate into neurons and release neurotrophic factors, facilitating neural repair in the penumbra and improving overall functional recovery [6, 7]. Notably, studies have clarified that NSCs can inhibit the adverse effects of Notch signaling by secreting GDNF following spinal cord injury [8]. Additionally, NSC transplantation promoted motor function recovery in stroke mice, mitigated post‐stroke neuroinflammation, and stimulated the proliferation of endogenous NSCs [9, 10]. Moreover, NSC transplantation was shown to promote angiogenesis in the rat brain following ischemia/reperfusion injury, an effect associated with enhanced vascular endothelial growth factor (VEGF) expression and increased microvessel density [11].

NSCs can be obtained from primary cells isolated from the subventricular zone (SVZ) of the fetal brain, as well as from induced pluripotent stem cells (iPSCs) and embryonic stem cells (ESCs). Following in vivo transplantation of human primary NSCs, researchers found that 93% of the cells differentiated into GFAP‐positive astrocytes, with only a limited fraction differentiating into neurons [12]. Meanwhile, approximately 20% of ESCs‐derived neural precursor cells transplanted in vivo differentiated into GFAP‐positive astrocytes [13, 14].

Despite the high therapeutic potential of NSCs, their clinical application in IS faces several challenges. First, the heterogeneity of NSCs sources and lineage‐specific differentiation propensities contribute to inconsistent outcomes after transplantation [15]. Second, precisely controlling the fate of transplanted NSCs remains difficult. Although NSCs can differentiate into neurons, astrocytes, and oligodendrocytes, the pathological microenvironment biases their differentiation toward astrocytic lineages, leading to glial scar formation and ultimately hindering functional recovery [16]. Since IS largely affects both neural and vascular components, therapeutic strategies should focus on neuronal preservation and restoration of vascular integrity and function. Vascular repair is crucial for the re‐establishment of cerebral blood flow (CBF), which maintains the integrity of the blood–brain‐barrier and rebuilds a supportive microenvironment that promotes neuronal survival and regeneration [1, 17]. This study aimed to assess whether non‐embryoid body (nEB)‐derived NSCs can promote vascular functional recovery after IS, as to provide a reliable translational strategy for stem cell therapies.

## 2 Materials and Methods

### 2.1 hESCs Differentiation via Embryoid Body (EB) Formation

Human embryonic stem cells (hESCs; Q‐CTS‐hESC‐2 cell line Q2) obtained from the Institute of Zoology, Chinese Academy of Sciences, were cultured until the colonies reached an appropriate size for differentiation. The cells were then digested with Dispase (17105‐041, Gibco, Thermo Fisher Scientific, Waltham, MA, USA) for 3 min, after which the clones were gently dissociated. The resulting embryoid bodies (EBs) were transferred to a low‐adsorption culture dish and incubated with 10–15 mL of neural induction medium (NIM) consisting of DMEM/F12 (11330‐032; Gibco), Neurobasal medium (21103‐049; Gibco), N2 supplement (17502‐048; Gibco), B27 supplement (17504‐044; Gibco), 2 mM GlutaMAX (35050‐061; Gibco), 10 μM SB431542 (1614; Tocris, Bio‐Techne, Japan), and 500 nM LDN193189 (04‐0074; Stemgent, Beltsville, MD, USA). The NIM was refreshed daily from day 1 through day 3. On day 4 of differentiation, EBs were transferred onto six‐well plates pre‐coated with recombinant human vitronectin (rhVTN, A14700; Gibco) at a moderate density. The cells were maintained in NIM with daily medium changes until day 9. From day 9 to day 10, the medium was replaced with neural preservation medium (NPM). On day 11, bFGF (100‐18B; Peprotech) was added to the NPM. The detailed components of the NIM and NPM are listed in Table S1.

### 2.2 hESCs Differentiation via Non‐Embryoid Body (nEB) Method

hESCs were dissociated using Accutase (A11105‐01; Gibco) and resuspended in E8 medium (A15169‐01, Gibco). The cells were then seeded at a density of 1.5 × 10^5^ cells/cm^2^ to ensure optimal attachment and growth conditions for subsequent neural induction. Differentiation was initiated by replacing E8 medium with a modified NIM composed of E6 medium (A15165‐01; Thermo Fisher Scientific) supplemented with 2 mM GlutaMAX, 500 nM LDN193189, and 10 μM SB431542 [18, 19]. From day 1 to day 12, cells were maintained in the same NIM, which was refreshed daily. After a 12‐day differentiation period, the cells were assessed for neural lineage specification.

For subsequent neuronal induction, nEB‐NSCs were washed with DPBS on day 10 and maintained in NPM medium for another 10 days with daily medium changes. On day 20, the cells were dissociated into a single‐cell suspension and replated at a density of 5 × 10^4^ cells per well in 6‐well plates. The NPM medium was supplemented with 10 μM DAPT (2634/10; Bio‐Techne, Minneapolis, USA), 100 μM AA (A4403; Sigma, USA), 20 ng/mL BDNF (450‐02; PeproTech, USA), and 20 ng/mL GDNF (450‐10; PeproTech). By day 22, the cells began to exhibit a neuronal morphology.

### 2.3 Differentiation and Tube Formation of Vascular Endothelial Cells

hESCs (mCherry‐H9 cell line) were digested into a single‐cell suspension and seeded onto new culture plates pre‐coated with 1% gelatin (G1890; Sigma) at a moderate density. Endothelial differentiation medium (CC‐4176; Lonza, Walkersville, MD, USA) was added, and the cells were maintained in culture for 6 days. Following differentiation, PE‐positive endothelial cell (EC) populations were enriched using flow cytometry and subsequently seeded onto new culture plates for further expansion and experimental procedures.

#### 2.3.1 Endothelial Cell Tube Formation Assay

96‐well plates were coated with GFR‐Matrigel (354230; Corning, Arizona, USA) and the matrix gel was allowed to solidify. Then, 50,000 ECs were inoculated per well, with three replicate wells per group. Images were captured between 8 and 16 h later. Two groups were established: (1) Blank: EC medium only; (2) nEB‐NSCs: nEB‐NSCs' overnight medium mixed with EC medium, which consisted of culture medium collected from nEB‐NSCs mixed with EC medium at a 1:1 ratio.

### 2.4 Flow Cytometry and Fluorescence Activated Cell Sorting

Cells were dissociated into single‐cell suspensions and fixed with Cytofix/Cytoperm solution (554722; BD Biosciences, USA). To assess the expression of neural markers, the cells were stained with antibodies against PAX6 and SOX2 for 30 min at room temperature. Following staining, 1 mL of DPBS was added, and the samples were centrifuged at 1200 rpm for 3 min. The supernatant was discarded, and the cells were resuspended in 300 μL of DPBS.

The flow cytometry antibodies used in this study are listed in Table S2. Flow cytometry analysis was performed using a BD Fortessa flow cytometer, with FlowJo software used for quantification. mCherry‐positive vascular ECs were sorted using a BD FACS Fusion cell sorter.

### 2.5 Quantitative Real‐Time PCR (RT‐PCR)

Total RNA was extracted from either the core region of IS‐induced mouse brain tissue or from adherent cultured cells using TRIzol Universal Reagent (A0717A01; Yeasen, Shanghai, China). RNA was reverse‐transcribed into cDNA following the protocol provided with the Hifair II 1st Strand cDNA Synthesis SuperMix for qPCR (11123ES60; Yeasen, Shanghai, China). RT‐PCR was performed using Hieff QPCR SYBR Green Master Mix (11184ES08; Yeasen) as the detection dye. All measurements were conducted in triplicate, and mRNA levels were normalized to *Gapdh* expression. The primer sequences used in this study are listed in Table S3.

### 2.6 Experimental Animals and Ethics Statement

Male C57BL/6 mice aged 8–10 weeks were procured from SiPeiFu Company (SiPeiFu, Beijing, China). Male animals were used to ensure consistency across the stroke model and reduce variability, given the known neuroprotective effects of estrogen [20, 21, 22, 23, 24, 25]. All animals were housed in a controlled environment with regulated temperature and humidity, maintained on a 12 h light–dark cycle. Water and food were supplied ad libitum. All animal experiments were approved by the Animal Care Committee of the Institute of Zoology (approval number: IOZ‐IACUC‐2021‐105) and conducted in accordance with ethical guidelines. Efforts were made to minimize animal suffering and reduce the number of animals used.

### 2.7 Transient MCAO (tMCAO) Model

Mice were anesthetized with tribromoethanol (Avertin; intraperitoneal injection, 400 mg/kg, T48402; Sigma‐Aldrich) and fixed on a surgical platform. The common carotid artery was exposed, and the external and internal carotid arteries were separated carefully. The distal end of the external carotid artery was ligated, while the proximal end was cut and notched. A silicon‐coated monofilament (Doccol Corporation) was inserted into the internal carotid artery and advanced to the middle cerebral artery (MCA) to induce ischemia [6]. After 60 min, the monofilament was slowly withdrawn to restore blood flow. Mice exhibiting a reduction of over 70% in cerebral blood flow in the temporal lobe, confirmed by laser speckle Imaging (RFLSI‐ZW; RWD Life Science Co. Ltd., Guangdong, China) during tMCAO, were selected for follow‐up studies. tMCAO predominantly damaged motor‐related regions, including the cerebral cortex and striatum (Figure S6A). The infarct volume showed ongoing evolution for 3 months (Figure S6B).

### 2.8 Cell Transplantation

Animals were randomly assigned to control and intervention groups, with the latter receiving neural stem cell transplants 14 days after tMCAO surgery, when the inflammatory cells in the brain had largely decreased (Figure S6C). Cell transplantation was performed at two sites─one in the cortex and one in the striatum─adjacent to the infarct zone, using the following coordinates: Anterior–Posterior (AP): +0.1 mm, Medial‐Lateral (ML): +1.5 mm, Dorsal‐Ventral (DV): −4.5 mm for the striatum, and AP: −1.0 mm, ML: +1.5 mm, DV: −1.5 mm for the cortex.

nEB‐NSCs were dissociated into a single‐cell suspension and maintained on ice prior to transplantation. Each injection site received 1 μL of the cell suspension (1 × 10^5^ cells), whereas control animals received 1 μL of sterile saline. Each mouse received a single transplantation 2 weeks after tMCAO surgery. The sacrifice time points for each outcome measure are presented in Figure 3.

### 2.9 Immunofluorescence

The mice were transcardially perfused and post‐fixed for 24 h, then dehydrated in a 30% sucrose solution for 72 h prior to frozen sectioning. Frozen slices were washed with 0.5% Triton X‐100 in DPBS. Primary and secondary antibodies were applied at room temperature for 2 h, and brain slices were mounted using DAPI Fluoromount (ZLI‐9557; Origene, Beijing, China). Consecutive brain sections were collected from each mouse to capture all graft‐containing regions guided by graft sites. The series covered the rostrocaudal extent of the infarct and graft, with at least two such sections in each group. Images were acquired at the same magnification and processed under identical settings, and cell numbers were quantified per area. Adherent cells were fixed using 4% PFA for 30 min, followed by three washes with DPBS before blocking and processing for immunofluorescence. Images were captured using a Zeiss LSM880 Fast Airyscan confocal microscope, and image analysis was performed with ImageJ and ZEAN software. Primary and secondary antibodies utilized in these experiments are listed in Tables S2 and S4, respectively.

### 2.10 Behavioral Tests

The open field test (OFT) and the cylinder test were conducted at 0, 1, 2, 3, 4, and 6 months after nEB‐NSCs grafting. All tests were conducted by an investigator blinded to the experimental conditions in a soundproof behavioral testing room with controlled light intensity, temperature, and humidity.

In the OFT, mice were gently placed in the center of the open field box, and total distance moved and speed were recorded over 10 min. In the cylinder test, mice were placed in a transparent cylinder, and their activities were recorded for 10 min. Forepaw contacts with the cylinder wallleft, right, or both—were recorded for each mouse, and the proportion of use on the injured (right) side was calculated.

### 2.11 Magnetic Resonance Imaging (MRI)

An 11.7 T MRI scanner (Bruker BioSpin, Billerica, MA, USA) was used to acquire T2‐weighted images (T2WI). In vivo scans were performed before nEB‐NSCs grafting and again 6 months after transplantation. The procedure followed these steps [26]: Mice were anesthetized with 1.5% isoflurane and positioned in the MRI scanner, then maintained under anesthesia with 1% isoflurane throughout the procedure. T2WI scan parameters were set as follows: field of view (FOV) = 20 × 16 mm, repetition time (TR)/echo time (TE) = 2800/30 ms, acquisition matrix = 256 × 205, 29 slices with a slice thickness of 0.5 mm, two mean values, and a Rapid Acquisition with Relaxation Enhancement factor = 8.

### 2.12 RNA Sequencing

The left cerebral cortex, striatum, and hippocampus of each mouse were isolated, snap‐frozen in liquid nitrogen for 2 min, and subsequently sent to BGI (Shenzhen, China) for RNA sequencing. Total RNA was extracted according to BGI's protocol, and RNA quality was assessed using a Fragment Analyzer.

### 2.13 Statistical Analysis

Statistical analyses were conducted using GraphPad Prism 9.0 (GraphPad Software, USA). For comparisons between two groups, a two‐tailed Student's *t*‐test was applied, while Tukey's multiple comparison test and one‐way analysis of variance were used for multiple group comparisons. All data are reported as the mean ± standard error of the mean. A *p*‐value < 0.05 was considered significant. Data were examined using the Shapiro–Wilk test, and for groups with small sample sizes, normality was additionally evaluated by inspection of Q–Q plots. All datasets exhibited a normal distribution.

## 3 Results

### 3.1 Differentiation and Characterization of nEB‐NSCs

**FIGURE 1:**
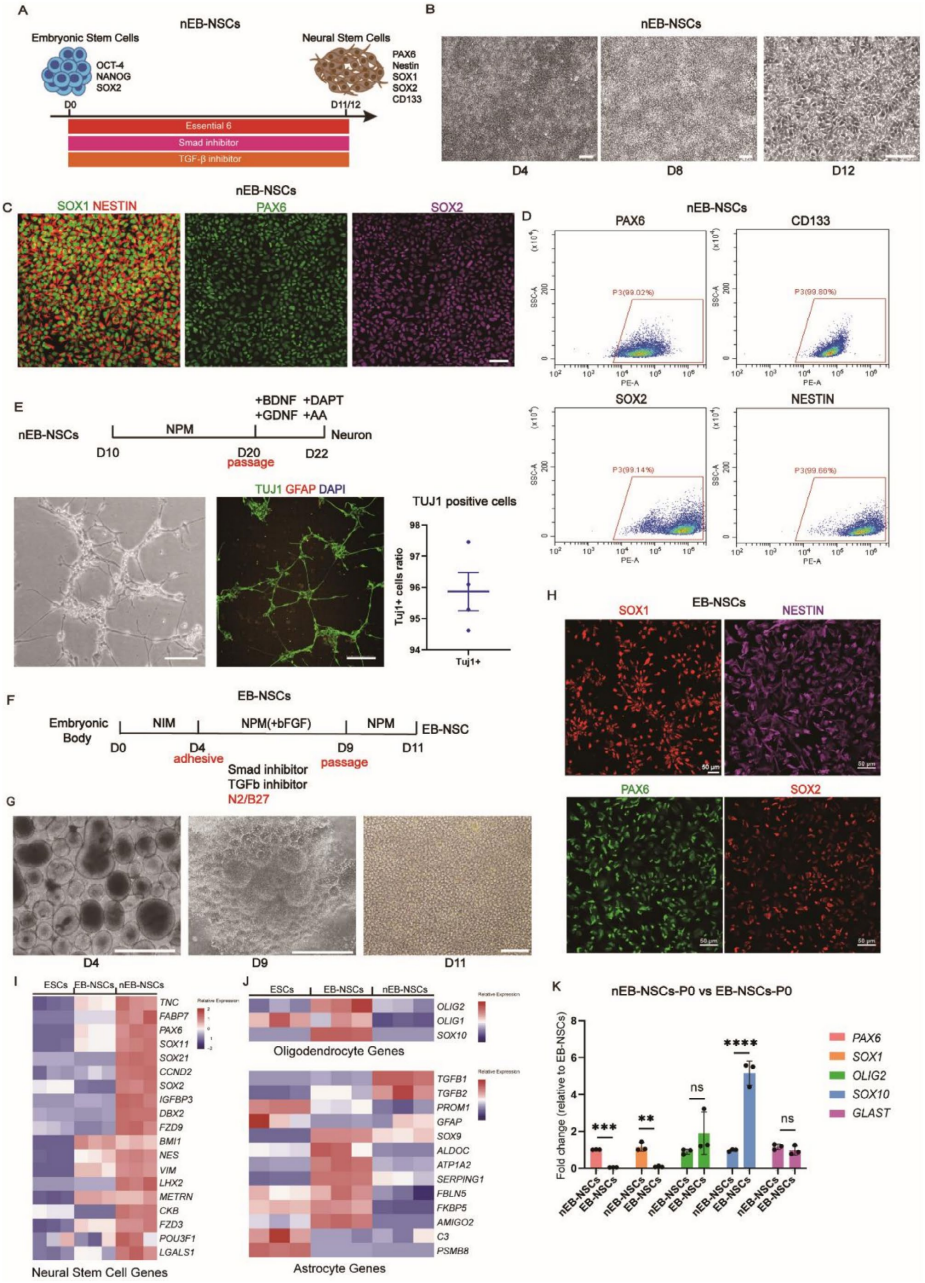
Identification and quality control of nEB‐NSCs obtained via the Essential 6 differentiation system. (A) nEB‐NSCs' differentiation pattern. (B) Dynamic changes in cellular morphology during differentiation progression. (C) Representative images of nEB‐NSCs at day 12, expressing the neural markers SOX1 and NESTIN (a), PAX6 (b), and SOX2 (c); scale bar = 50 μm. (D) Quantitative assessment of NSC differentiation through flow cytometric analysis of the expression of neural markers PAX6, SOX2, CD133, and Nestin. (E) Pattern of neuronal differentiation in nEB‐NSCs, with neuronal morphology (a) and expression of the neuronal marker TUJ1 (b) evidenced at day 22; *n* = 4, scale bar = 100 μm. (F) Representation of EB‐NSCs' differentiation pattern. (G, H) Cell morphology (G: A‐b, scale bar = 500 μm; c, scale bar = 100 μm), and identification of EB‐NSCs (H: Scale bar = 50 μm). (I) Heatmap showing the relative expression of NSC marker genes. (J) Heatmap displaying the relative expression of oligodendrocyte and astrocyte marker genes. (K) Analysis of neural cell genes and glia cell genes expression by RT‐PCR.

We assessed two strategies for deriving transplantable NSCs: one utilizing embryoid body (EB) formation, and the other following a direct differentiation protocol without EB formation (non‐EB) [27, 28]. Both strategies mimic embryonic developmental cues by incorporating inhibitors of the SMAD and TGF‐β signaling pathways (LDN193189 and SB431542, respectively) (Figure 1A). The NSCs derived from the non‐EB differentiation protocol were designated as nEB‐NSCs.

During differentiation, cells adhered to the culture surface as a monolayer with uniform morphology and small, evenly spaced intercellular gaps (Figure 1B). Immunofluorescence staining confirmed the expression of the NSC markers PAX6, SOX1, SOX2, and NESTIN (Figure 1C). To meet transplantation criteria, a standardized quality control workflow was established. Flow cytometry results further verified the high expression of PAX6, SOX2, NESTIN, and CD133, supporting their NSC identity (Figure 1D). Upon neuronal induction, nEB‐NSCs differentiated into TUJ1‐positive neurons, with no detectable GFAP‐positive astrocyte differentiation (Figure 1E).

For comparison, NSCs were also derived using an alternative differentiation system via the EB stage (EB‐NSCs). Similar to the nEB‐NSCs protocol, this method employed inhibitors targeting the SMAD and TGF‐β signaling pathways (Figure 1F). Cells were digested into clusters to form EBs, cultured under adherent conditions from day 4 to day 9 to induce rosette formation, then digested again before being collected on day 11 for characterization and subsequent transplantation (Figure 1G). The resulting EB‐NSCs expressed key NSCs markers, including SOX1, PAX6, SOX2, and NESTIN, confirming their NSCs identity (Figure 1H).

### 3.2 nEB‐NSCs Exhibit Enhanced Neuronal Fate Commitment and Functional Potential In Vitro

We performed transcriptome sequencing on NSCs derived from both differentiation systems, and the results confirmed that both EB‐NSCs and nEB‐NSCs originated from ESCs (Figure S1A,B). Compared to EB‐NSCs, nEB‐NSCs showed significantly higher expression of NSC‐related genes, including *PAX6, SOX2, VIM, TNC, SOX21, SOX11*, and *NES* (Figure S1C). Conversely, there was a significant reduction in the expression of oligodendrocyte markers (*OLIG1, OLIG2, SOX10*) and astrocyte markers (*SOX9, ALDOC, ATP1A2, SERPING1, AMIGO2*) (Figure 1I,J). These results indicate that nEB‐NSCs are more strongly committed to the neuronal lineage, with a diminished capacity for glial lineage commitment.

Importantly, nEB‐NSCs upregulated several genes involved in angiogenesis, including *CLDN5*, *CDH5*, *SOX7*, *EDH4*, *CEACAM1*, and *PECAM1* (Figure S1D,F). Differential gene analysis revealed that nEB‐NSCs were significantly enriched in pathways associated with vascular regeneration, neurogenesis, neural stem cell proliferation, neuron projection guidance, and neuronal synapse formation (Figure S1E,G). These findings suggest that nEB‐NSCs possess enhanced capabilities in promoting both vascular and nerve regeneration.

**FIGURE 2:**
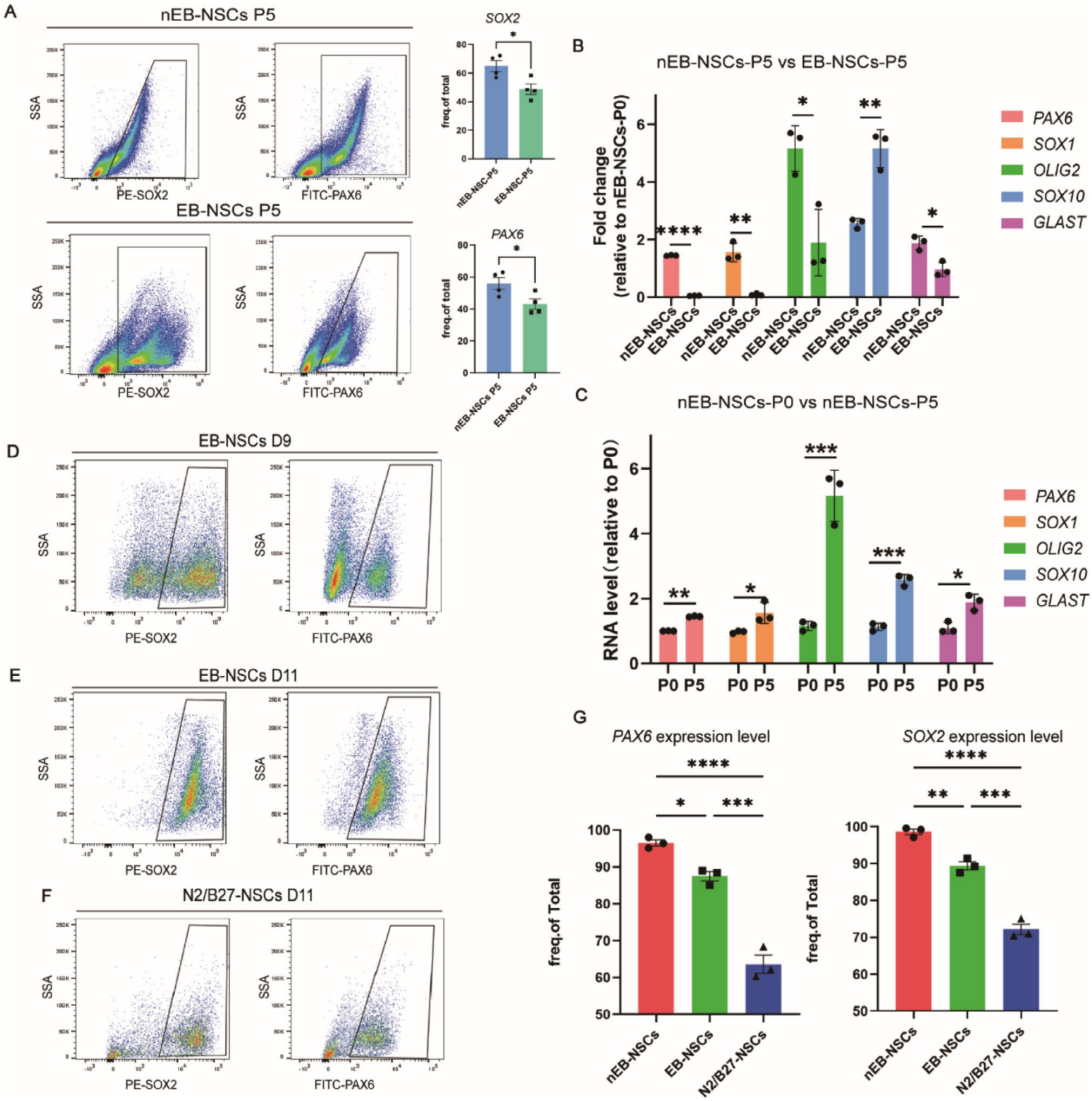
Comparison of the efficiency of the NSC differentiation systems. (A) Flow cytometry plots and quantification of PAX6 and SOX2 expression in nEB‐NSCs and EB‐NSCs passaged 5 times. (B) RT‐PCR analysis of neuronal and oligodendrocyte markers in nEB‐NSCs (nEB‐NSCs‐P5) and EB‐NSCs passaged five times (EB‐NSCs‐P5). (C) RT‐PCR analysis highlighting differences in the expression of neural and glial markers in nEB‐NSCs at P0 and P5. (D, E) Flow cytometry data plots demonstrating increased expression of neural markers in EB‐NSCs following passage at day 9. (F) Flow cytometry analysis showing the proportion of PAX6 positive and SOX2 positive cells observed in the total cell population derived from the N2/B27 differentiation system. (G) Flow cytometry analysis depicting the distribution of PAX6‐positive and SOX2‐positive nEB‐NSCs, EB‐NSCs, and NSCs derived from the N2/B27 differentiation system (N2/B27‐NSCs).

To investigate the differences between the two differentiation systems, we analyzed the expression of key lineage markers, including *PAX6*, *OLIG2*, and *SOX10*. Compared to EB‐NSCs, nEB‐NSCs exhibited significantly higher expression of *PAX6* and lower expression of *SOX10* (Figure 1I,J). To examine the cause of these differences, we considered the purification process. Notably, after rosette formation, EB‐NSCs require passaging enrichment to achieve more than 90% purity to meet transplantation criteria (Figure 2A,B). In contrast, nEB‐NSCs do not require passaging and can be used for transplantation after 12 days of differentiation. Based on this, we hypothesized that the passaging process may be the primary factor contributing to the observed differences. Therefore, we passaged both types of NSCs up to five times and collected cells at different passages for gene expression analysis. Under this five‐passage protocol, the expression of glial cell transcription factors, including OLIG2, SOX10, and GLAST, increased significantly (Figure 1K). Concurrently, the proportion of PAX6‐ and SOX2‐positive nEB‐NSCs and EB‐NSCs decreased by passage 5 (P5) (Figure 2C).

In addition, compared to non‐passaged nEB‐NSCs (nEB‐NSCs‐P0), passaged EB‐NSCs (EB‐NSCs‐P5) exhibited reduced expression of neuronal markers such as PAX6 and SOX1, alongside increased expression of the oligodendrocyte marker SOX10. The expression levels of OLIG2 and GLAST remained relatively stable (Figure 2D). Similarly, in nEB‐NSCs, passaging to P5 led to a significant upregulation of OLIG2, SOX10, and GLAST compared to P0 cells (Figure 2E). These findings indicate that repeated passaging downregulated neuronal marker expression while enhancing glial lineage marker expression, thereby shifting the differentiation bias of NSCs toward a glial fate. Based on these results, we recommend minimizing passaging during differentiation to better preserve the neuronal identity of NSCs.

In addition to assessing passaging effects, the two NSCs types were cultured under distinct conditions. To assess the influence of the culture system, we examined a protocol in which ESCs were directly differentiated without EB formation, using medium supplemented with N2 and B27. Differentiation efficiency was evaluated after 12 days. Flow cytometry analysis showed that the proportions of PAX6‐ and SOX2‐positive cells were lower (Figure 2F) and significantly reduced compared to both EB‐NSCs and nEB‐NSCs (Figure 2G).

### 3.3 Grafted nEB‐NSCs Adopt a Neuronal Fate in the Ischemic Brain

**FIGURE 3:**
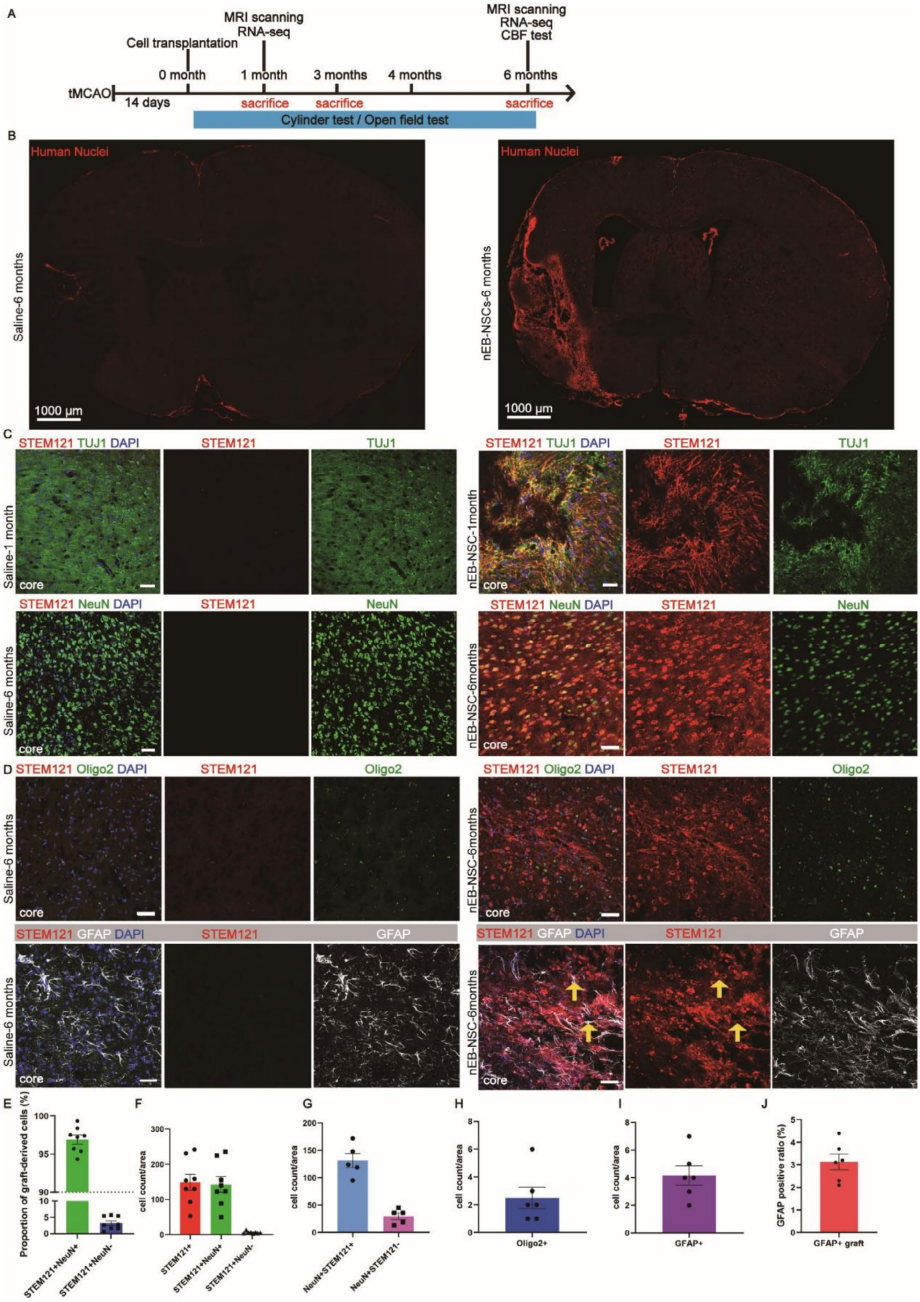
nEB‐NSCs survive and differentiate into neurons in IS mice. (A) Schematic diagram of the nEB‐NSCs transplantation process. (B) Representative images of HuNu (human nuclei) expression in nEB‐NSCs 6 months after transplantation; scale bar = 1000 μm. (C) Representative immunofluorescence images of STEM121 and TUJ1 expression 1 month after transplantation and of STEM121 and NeuN expression 6 months after transplantation in the Saline and nEB‐NSCs groups; scale bar = 50 μm. (D) Representative immunofluorescence images of STEM121 along with oligodendrocyte (Oligo2) and astrocyte (GFAP) markers in the Saline and nEB‐NSCs groups; scale bar = 50 μm. (E, F) Number and proportion of transplanted neuronal (STEM121^+^/NeuN^+^) and non‐neuronal (STEM121^+^/NeuN^−^) cells 6 months post‐transplantation; *n* = 8. (G) Quantification of neuronal populations 6 months post‐transplantation, including transplanted nEB‐NSCs exhibiting neuronal identity (NeuN^+^/STEM121^+^) and host‐derived neurons (NeuN^+^/STEM121^−^); *n* = 5. (H, I, J) Quantification of oligodendrocyte (Oligo2) and astrocyte (GFAP) differentiation status in the Saline and nEB‐NSCs groups; *n* = 6.

To evaluate the differentiation capacity of nEB‐NSCs in vivo, we transplanted them into the brains of mice subjected to IS, with cell injections performed 14 days after tMCAO surgery (Figure 3A). Six months after transplantation, human nuclei (HuNu)‐positive transplanted cells were found in the infarct zone, and were absent in infarcted tissue from saline‐injected (control) mice (Figure 3B). One month after transplantation, the majority of STEM121‐positive grafts around the infarct zone exhibited expression of the neuronal marker TUJ1 (Figure 3C). At this time point, there were still some STEM121‐positive grafts around the infarct zone expressing PAX6 (Figure S2A), indicating the continued presence of NSCs at this stage. By three months and six months post‐transplantation, STEM121‐positive grafts expressed the mature neuronal marker NeuN (Figure S2B,C), indicating their differentiation into mature neurons, where as by 6 months, 96.9% ± 1.6% of STEM121‐positive grafts co‐expressed NeuN (Figure 3C,E,F). Additionally, some NeuN^+^ STEM121^−^ host neurons were observed (Figure 3G). These findings suggest that nEB‐NSCs primarily differentiated into neurons within the pathological environment. No cells co‐expressing Oligo2/STEM121 were detected, indicating that nEB‐NSCs did not adopt an oligodendrocyte fate (Figure 3D,H). Moreover, only a small subset of STEM121‐positive cells co‐expressed GFAP, accounting for 3% ± 0.35% (Figure 3D,I,J), reflecting a limited tendency toward astrocytic differentiation.

In contrast, after transplantation, EB‐NSCs expressed both the neuronal marker TUJ1 and the astrocyte marker GFAP, indicating that a subset of the transplanted cells had differentiated into astrocytes, accounting for 8% ± 0.87% (Figure S3A,B). These findings suggest that, compared to EB‐NSCs, nEB‐NSCs exhibit a stronger commitment to the neuronal lineage.

### 3.4 Grafted nEB‐NSCs Exhibit Region‐Specific Neuronal Identities

**FIGURE 4:**
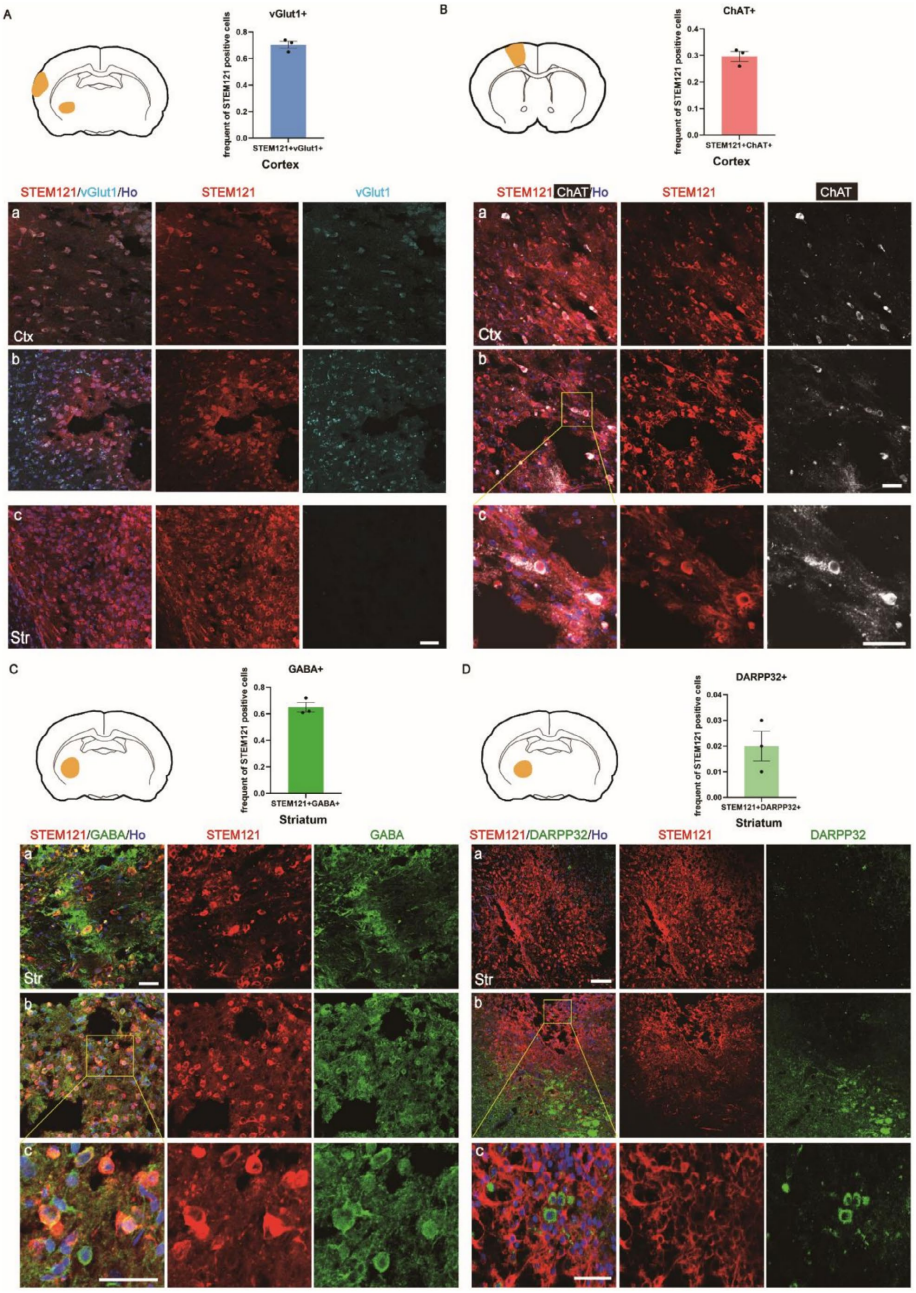
Grafted nEB‐NSCs exhibit region‐specific neuronal phenotypes 6 months after transplantation. (A) Glutamatergic neurons in the cortex were identified by vGlut1 immunolabeling (cyan) (Ctx, a,b). The bar graph quantifies the fraction of STEM121‐positive cells co‐expressing vGlut1 (*n* = 3 for each group); scale bar = 50 μm. (B) Motor neurons in the cortex were identified by ChAT immunolabeling (white). The corresponding bar graph shows the proportion of STEM121‐positive cells co‐expressing ChAT (*n* = 3 for each group); scale bar = 50 μm. (C) GABAergic neurons in the striatum (Str) were identified by GABA immunolabeling (green). The quantification of STEM121^+^/GABA^+^ cells is shown in the bar graph (*n* = 3 for each group); scale bar = 50 μm. (D) Medium spiny neurons in the striatum were identified by DARPP32 immunolabeling (green). The bar graph presents the percentage of STEM121^+^ cells co‐expressing DARPP32 (*n* = 3 for each group); scale bar = 100 μm. These analyses were performed 6 months after transplantation. All statistical analyses were performed using images acquired under the same magnification.

Given that glutamatergic neurons are the predominant excitatory neuronal subtype in the cortex, we first evaluated differentiation into this lineage 6 months post‐transplantation. STEM121‐positive grafted cells located near the infarcted cortical area co‐expressed the vesicular glutamate transporter vGlut1, identifying them as glutamatergic neurons (Figure 4A‐a,b). Quantitative analysis showed that 70.33% ± 2.72% of the grafted cells were positive for both STEM121 and vGlut1. In contrast, no vGlut1‐positive grafted cells were detected in the striatum (Figure 4A‐c). Given the presence of cholinergic neurons in the motor cortex, we assessed cholinergic differentiation. A subset of the surviving transplanted cells expressed choline acetyltransferase (ChAT), suggesting differentiation into choline'rgic neurons (Figure 4B), with 29.67% ± 3.22% of STEM121‐positive cells co‐expressing ChAT.

The striatum is composed predominantly of GABAergic medium spiny neurons, which account for approximately 95% of striatal neurons, while the remaining 5% are spineless interneurons [29]. To determine whether the transplanted cells in the striatum adopted a GABAergic identity, we assessed GABA expression. Quantification showed that 65.00% ± 6.08% of STEM121‐positive cells co‐expressed GABA, indicating successful differentiation into GABAergic neurons and potential contribution to basal ganglia circuit repair (Figure 4C). However, only 2.00% ± 0.57% of STEM121‐positive cells expressed DARPP32, suggesting that only a small fraction of grafted cells differentiated into striatal projection neurons (Figure 4D).

### 3.5 nEB‐NSCs Transplantation Promotes Neuronal Regeneration and Vascular Development

To evaluate neuronal repair in the ischemic brain, we performed transcriptomic analyses of brain tissue from the infarcted hemisphere at 1 and 6 months following transplantation. One month post‐transplantation, compared to the saline group, genes upregulated in the nEB‐NSCs group were primarily enriched in pathways related to positive regulation of neurogenesis, neural projection development, neural progenitor cell differentiation, and inhibition of neuronal apoptosis (Figure S4A,B). In contrast, downregulated genes were mainly associated with neurotransmitter receptor activity and neurotransmitter transport (Figure S4B). Previous studies have identified excitotoxicity caused by excessive neurotransmitter release as a key pathological mechanism in IS [30, 31]. Our findings suggest that nEB‐NSCs transplantation may alleviate neurotransmitter‐induced excitotoxicity, highlighting its therapeutic potential in the treatment of IS.

Six months post‐transplantation, compared to the saline group, the upregulated genes in the nEB‐NSCs group were significantly enriched in pathways associated with central nervous system (CNS) development and neuronal regeneration. These included telencephalic forebrain development, neuronal differentiation, neuronal fate specification, CNS neuron differentiation, and axon guidance (Figure S4C). This enrichment pattern highlighted the critical role of nEB‐NSCs transplantation in promoting CNS repair and neuronal regeneration. Furthermore, gene network analysis showed that a broad range of development‐related pathways shared common genes, indicating a coordinated regulatory relationship and potential crosstalk among these processes (Figure S4D).

**FIGURE 5:**
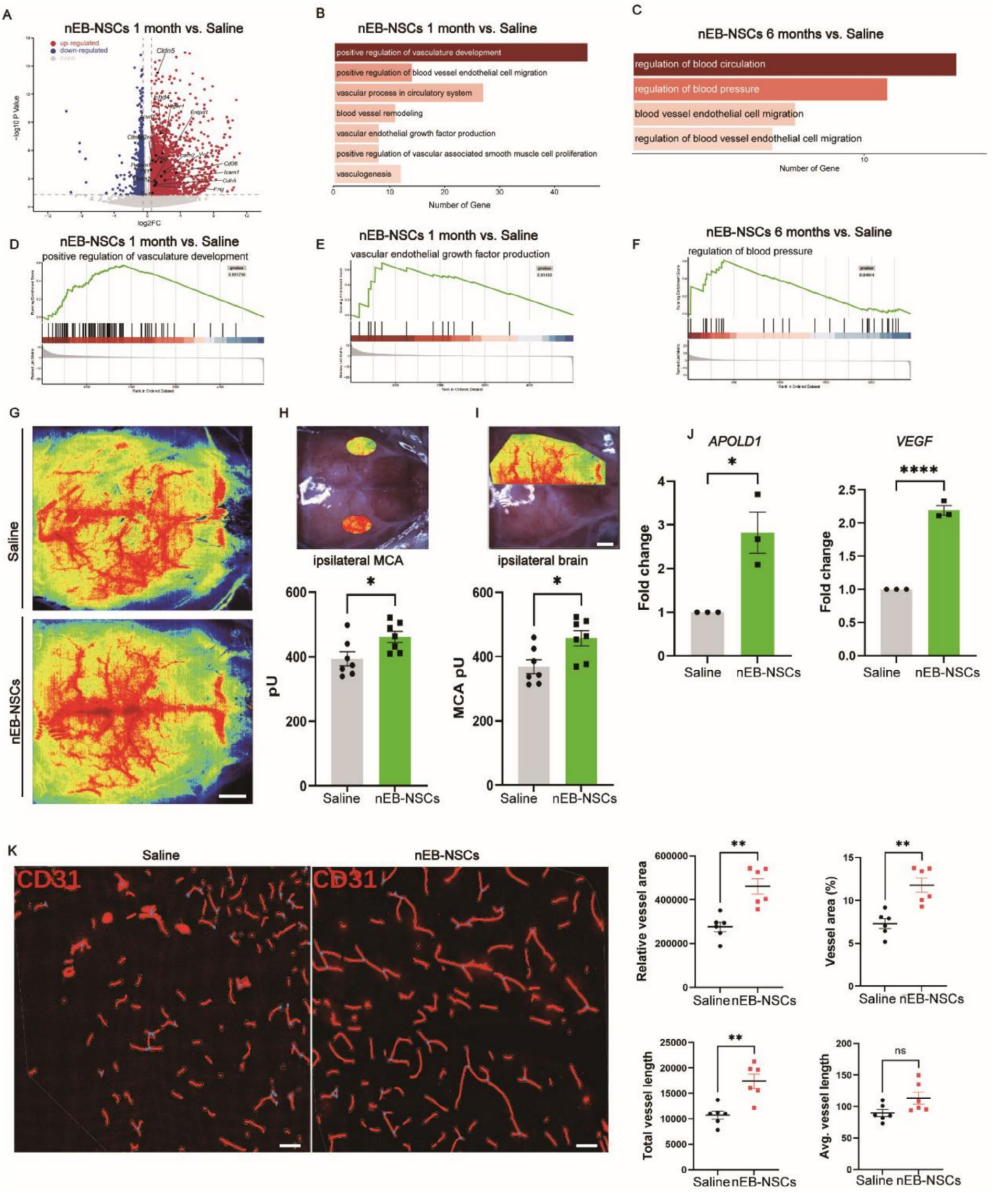
nEB‐NSCs transplantation promotes vascular function recovery. (A) Volcano plot showing DEGs between the nEB‐NSCs and Saline groups at 1 month post‐transplantation. Significantly upregulated and downregulated genes (*p* < 0.05, |log_2_FC| > 1) are shown in red and blue, respectively. (B) Bar plot of GO analysis for upregulated DEGs between the nEB‐NSCs and Saline groups at 1 month post‐transplantation. The top enriched biological processes related to neurogenesis are displayed, along with corresponding −log_10_ *p* values and gene numbers. (C) Bar plot for GO analysis of upregulated DEGs between the nEB‐NSCs and Saline groups at 6 months post‐transplantation. The top enriched biological processes related to the vasculature are displayed, along with corresponding −log_10_ *p* values and gene numbers. (D, E) GSEA of positive regulation of vasculature development between the nEB‐NSCs and Saline groups at 1 month post‐transplantation. (F) GSEA of regulation of blood pressure between the nEB‐NSCs and Saline groups at 6 months post‐transplantation. (G–I) CBF measurements in nEB‐NSCs/Saline graft mice. Left middle arterial blood perfusion (G), as well as blood perfusion in the damaged hemisphere (I), were significantly higher in the nEB‐NSCs group than in the control group (*p* < 0.05; *n* = 7 for each group). (J) RT‐PCR analysis showing higher levels of the angiogenesis markers VEGF and APOLD1 in nEB‐NSCs graft mice (*p* < 0.05; scale bar = 100 μm; *n* = 3 for each group). (K) Immunostaining of the blood vessel marker CD31 (red) and nuclear staining (Hoechst 33342, blue); scale bar = 50 μm. Quantification revealed that vessel area, vessel area percentage, and total and average vessel length were significantly higher in the nEB‐NSCs group compared to the control group (*p* < 0.05). Data were analyzed using AngioTool software. For each group, three animals were analyzed, and two independent brain slices were selected per animal for quantification (*n* = 6 sections per group).

IS results from sudden arterial blockage, leading to ischemia and hypoxia affecting neurons and ECs. The role of NSCs in vascular repair remains unclear; thus, we investigated whether NSCs transplantation promotes vascular recovery after ischemic injury. One month post‐transplantation, several vascular‐associated genes, including *Cdh1*, *Cdh5*, and *Pecam1*, were significantly upregulated in the nEB‐NSCs group (Figure 5A), indicating enhanced vascular development. GO enrichment analysis further indicated strong associations with vascular development, vascular remodeling, angiogenesis, and VEGF production (Figure 5B). Additionally, GSEA indicated a significant positive correlation in the regulation of VEGF production and vascular development after nEB‐NSCs transplantation (Figure 5D,E).

Six months post‐transplantation, GO analysis revealed sustained upregulation of genes involved in blood circulation, blood pressure regulation, and vascular endothelial cell migration (Figure 5C). GSEA also showed significant positive enrichment of pathways regulating blood pressure in the nEB‐NSCs group (Figure 5F). Together, these results suggest that vascular reconstruction is initiated early after transplantation and continues over an extended period, contributing to long‐term vascular recovery.

### 3.6 nEB‐NSCs Transplantation Promotes Vascular Function Recovery

Because vascular restoration is a prolonged and dynamic process, functional improvements often lag behind transcriptional changes. Therefore, we evaluated vascular integrity and function at 6 months post‐transplantation. In the nEB‐NSCs group, laser speckle contrast imaging showed that CBF in the left MCA territory was significantly higher than that in the saline group (Figure 5G,H). Moreover, total CBF in the ischemic hemisphere was significantly increased (Figure 5G,I). In addition, RT‐PCR analysis revealed that *VEGFA* and *APOLD1*, two genes with important roles in angiogenesis, were significantly upregulated in the nEB‐NSCs group (Figure 5J). Quantification of cortical vascular density further demonstrated that both vessel length and vessel area were significantly increased in the nEB‐NSCs group (Figure 5K). Moreover, in vitro tube formation assays demonstrated that nEB‐NSCs enhanced the ability of ECs to form vascular structures. Quantification showed a significant increase in vascular branches, vascular density, and total vascular area in the nEB‐NSCs group compared to controls (Figure S5A–C).

Integrating transcriptomic profiles with CBF measurements, we observed that vascular function was markedly improved in the nEB‐NSCs group. The high expression of genes related to blood circulation and EC migration indicates enhanced vascular regeneration and restored function after transplantation. Collectively, these findings indicate that nEB‐NSCs not only promoted vascular regeneration but also enhanced vascular function to improve CBF following transplantation.

### 3.7 nEB‐NSCs Transplantation Reduces Infarct Volume and Promotes Behavioral Recovery

**FIGURE 6:**
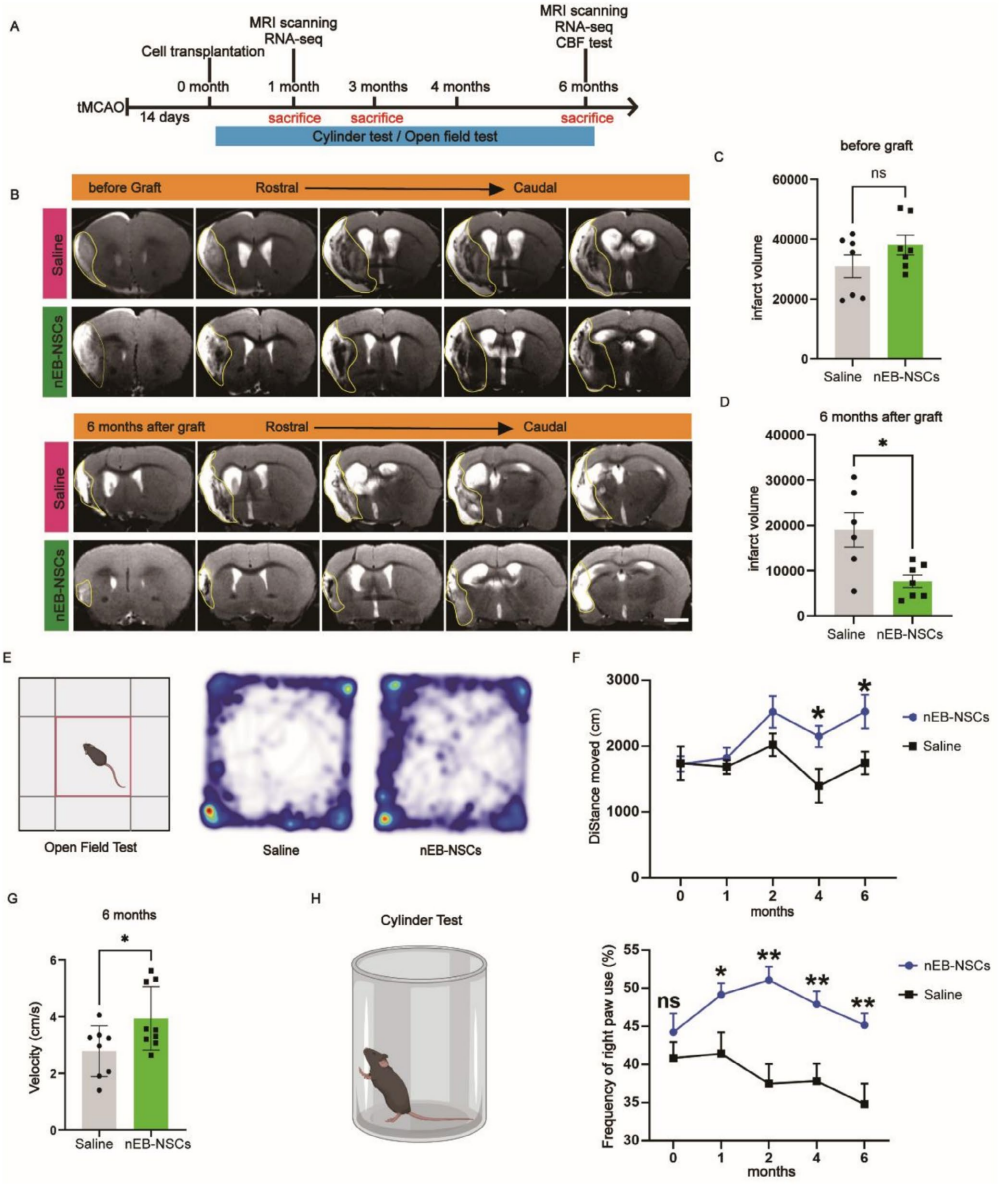
nEB‐NSCs transplantation reduces infarct volume and promotes behavioral recovery. (A) Experimental design. (B) MRI images showing infarct volume in the nEB‐NSCs and Saline (control) groups. (C, D) Quantification of infarct volume prior to and at 6 months following nEB‐NSC transplantation; scale bar = 1 mm; *p* < 0.05; *n* = 7 mice per group (C), *n* = 6 for the Saline group and *n* = 7 for the nEB‐NSCs group (D). (E–G) Schematics and summary results of monthly locomotor testing (OFT). (H) Schematics and summary results of monthly forelimb asymmetry assessment (cylinder test). In both behavioral tests, *n* = 8 for the nEB‐NSCs group and *n* = 9 for the saline group; *p < 0.05, **p < 0.01.

Prior to transplantation, animals were randomly and evenly assigned to two groups based on infarct volume assessed by MRI. Detailed information regarding sacrifice time points and outcome measures is presented in Figure 6A. Six months post‐transplantation, MRI analysis revealed that the infarct volume was notably reduced in the nEB‐NSCs group compared to the saline‐injected control group, indicating that nEB‐NSCs transplantation effectively limited infarct progression (Figure 6B–D).

To evaluate functional recovery, we conducted longitudinal behavioral tests including the OFT and cylinder test. In the OFT, the nEB‐NSCs group exhibited a significant increase in motor activity after 4 and 6 months, with mean distances of 2150.659 ± 559.673 cm and 2527.408 ± 808.500 cm, respectively. These values were significantly higher compared to the control group, for which the moved distances were 1399.362 ± 727.672 cm and 1746.393 ± 486.586 cm at the respective time points (Figure 6E,F). Further indicating improved motor performance, by 6 months post‐transplantation, mice in the nEB‐NSCs group moved at an average speed of 3.948 cm/s, which was significantly higher than the 2.788 cm/s recorded in the saline group (Figure 6G).

In the cylinder test, at all time points post‐transplantation, the frequency of using the right forelimb was significantly higher in the nEB‐NSCs group than in the saline group by 6 months (Figure 6H), suggesting reduced limb injury and significantly enhanced motor recovery following nEB‐NSCs transplantation.

## 4 Discussion

NSC transplantation is a promising therapeutic strategy for IS. However, the tendency toward glial differentiation remains a major barrier to effective cell therapy. In this study, we identified a neural induction system that promotes neuronal lineage commitment and evaluated its in vivo efficacy in comparison with a conventional differentiation method commonly used for transplantation.

First, in vitro, nEB‐NSCs exhibited markedly higher expression of neuronal markers relative to oligodendrocyte and astrocytic markers. Moreover, nEB‐NSCs showed enhanced capabilities in promoting neurogenesis, CNS neuron differentiation, and angiogenesis. The enhanced neuronal differentiation observed in nEB‐NSCs is attributable to the strategic selection of induction factors and the optimization of the differentiation protocol. SB431542 and LDN193189, key components of the neural induction system hereby tested, are well‐characterized modulators of neurogenic signaling pathways and drive neuronal fate commitment [32]. Compared to the EB‐based approach, nEB‐NSCs received a longer induction period of 12 days, formed a tightly adherent monolayer, and were not subjected to passaging during differentiation. These conditions minimized variability in factor exposure and limited glial lineage commitment, which typically requires extended culture and repeated passaging. Consistent with this, our data showed that passaging increased glial marker expression, whereas non‐passaged nEB‐NSCs maintained a more defined neuronal identity. Transcriptomic analysis confirmed this bias, with results showing high expression of neuronal lineage regulators such as FOXG1, GSX2, and EOMES [33, 34], which are critical for neurogenesis and CNS development. Gene enrichment analysis also showed that genes upregulated in nEB‐NSCs are involved in neuronal development and differentiation. Collectively, these findings proved that the proposed strategy, using defined neuronal induction, promotes neuronal lineage specification in nEB‐NSCs.

Second, some studies have proven that NSCs can promote angiogenesis. For example, a study showed that NSCs were able to improve RECA1^+^ blood vessel density and increased branching numbers [9], augmenting also VEGF secretion from both NSCs and astrocytes [11]. Moreover, the proliferation of collagen‐positive ECs was shown to be promoted by NSCs [35] However, the impact of NSCs on vascular function has rarely been investigated. In our study, we found that nEB‐NSCs not only improved brain microvessel density but also enhanced CBF, indicating functional recovery. Additionally, we observed robust upregulation of APOLD1, an early‐response endothelial protein induced by ischemia [36]. Previous studies have found that APOLD1 was able to promote vascular regeneration in the adult ischemic brain and help regulate endothelial function [37, 38, 39]. APOLD1 deficiency impairs PI3K/Akt signaling and enhances platelet reactivity, further emphasizing its function in vascular integrity as well as its therapeutic targeting in vascular disorders [37]. These findings raise the possibility that nEB‐NSCs may enhance angiogenesis not only through VEGF pathways but also via APOLD1‐mediated mechanisms. Future investigations should evaluate the function of APOLD1 in nEB‐NSCs induced vascular regeneration, potentially revealing a novel mechanism for post‐stroke recovery.

Despite the strengths of this work, some limitations remain. First, we did not explore the specific role of EB formation in glial specification, nor the molecular mechanisms underlying this glial bias. Future work will focus on defining the pathways regulating SOX10 expression and the influence of EB structure on glial differentiation. Second, although some neural markers were highly expressed in nEB‐NSCs, their roles in driving neuronal fate bias in vivo remain unclear. In future work, we plan to combine in vivo cell tracing with barcode labeling to track individual transplanted cells, aiming to characterize neural developmental factor expression patterns and examine cell–cell interactions at the single‐cell level. Third, we did not directly assess the structural or functional integration of transplanted cells into host neural circuits. Future studies will evaluate the functional integration of transplanted cells by directly assessing neural circuit restoration and single‐neuron excitability.

## 5 Conclusions

We applied and compared multiple differentiation approaches and identified a stable, uniform method for generating NSCs suitable for transplantation. The resulting nEB‐NSCs exhibited a pronounced bias toward neuronal lineage differentiation, prompting further investigation into the underlying mechanisms driving this fate. To ensure reproducibility, we also established a standardized quality control system using a flow cytometry workflow. Notably, beyond their capacity to replenish neurons, nEB‐NSCs enhanced functional recovery of the cerebral vasculature during the chronic phase of stroke. This dual functionality addresses a critical challenge in IS therapy and provides a solid foundation for clinical translation.

## Author Contributions

T.Z., B.H., Y.W.: study conception and design; T.Z.: experiment implementation and data analysis; D.L., Q.W.: tMCAO surgery operation; Q.G.: bulk RNA‐seq data analysis; T.Z., B.A.: animal care and behavioral tests; T.Z.: manuscript draft; Y.W., B.H.: manuscript revision. All authors contributed to the manuscript read and approved the final manuscript.

## Funding

This work was supported by the Strategic Priority Research Program of the Chinese Academy of Sciences, XDC0200000; Beijing Natural Science Foundation, Z230011; the National Key Research and Development Program of China, 2024YFA1107500.

## Ethics Statement

The study was approved by the ethical review board of the Institute of Zoology (approval number: IOZ‐IACUC‐2021‐105).

## Conflicts of Interest

The authors declare no conflicts of interest.

## Supporting information


**Table S1:** Detailed formulation of NIM and NPM.
**Table S2:** Antibodies used for immunofluorescence and fluorescence‐activated cell sorting (FACS).
**Table S3:** Primer sequences used for RT‐PCR.
**Table S4:** Secondary antibodies used in this study.
**Figure S1:** nEB‐NSCs exhibit higher neuronal differentiation and functional repair potential compared with EB‐NSCs. (A) PCA analysis of ESCs, EB‐NSCs, and nEB‐NSCs (*n* = 3). (B) Heatmap depicting the relative expression of ESC marker genes. (C) Heatmap showing genes associated with CNS expression among ESCs, EB‐NSCs, and nEB‐NSCs. (D) Volcano plot showing DEGs between EB‐NSCs and nEB‐NSCs. Significantly upregulated and downregulated genes (*p* < 0.05, |log_2_FC| > 1) are shown in red and blue, respectively. NSCs marker genes were specifically labeled. (E) Bar plot for GO analysis of upregulated DEGs between EB‐NSCs and nEB‐NSCs. The top enriched biological processes related to the vasculature are displayed, along with corresponding −log_10_ *p* values and gene numbers. (F) Volcano plot showing DEGs between EB‐NSCs and nEB‐NSCs. Significantly upregulated and downregulated genes (*p* < 0.05, |log_2_FC| > 1) are indicated in red and blue, respectively. Vasculature marker genes are specifically labeled. (G) Bar plot for GO analysis of upregulated DEGs between EB‐NSCs and nEB‐NSCs. The top enriched biological processes related to neurogenesis are displayed, along with corresponding −log_10_ *p* values and gene numbers.
**Figure S2:** Transplanted nEB‐NSCs exhibit a neuronal fate and express mature neuronal markers. (A) Representative images 1 month post‐transplantation showing nEB‐NSCs expressing the neural marker PAX6 (green), the human‐specific transplantation marker STEM121 (red), and nuclear counterstain DAPI (blue); scale bar = 20 μm. (B) Representative images 3 months post‐transplantation showing nEB‐NSCs expressing the mature neuronal marker NeuN (green) and STEM121 (red); scale bar = 50 μm. (C) Representative images 6 months post‐transplantation showing nEB‐NSCs expressing the mature neuronal marker NeuN (green) and STEM121 (red); scale bar = 50 μm.
**Figure S3:** EB‐NSCs transplanted into ischemic stroke mice differentiate into both neuronal and astrocytic lineages. (A) Immunostaining and quantification of EB‐NSCs expressing the immature neuronal marker TUJ1 and the human‐specific transplantation marker STEM121. Nuclei are stained with DAPI. (B) Immunostaining and quantification of EB‐NSCs expressing the astrocyte marker GFAP (white) and STEM121 (red). Nuclei are stained with DAPI. Brain slices were collected at 7 days post‐transplantation: *n* = 4.
**Figure S4:** nEB‐NSCs transplantation facilitates nerve regeneration in the ischemic brain. (A) Heatmap depicting the relative expression of neurogenesis‐related genes between nEB‐NSCs grafted for 1 month and saline‐injected control samples. (B) Bar plot for GO analysis of upregulated DEGs (top panel) and downregulated DEGs (bottom panel) between nEB‐NSCs grafted for 1 month and saline‐injected control samples. The top enriched biological processes related to neurogenesis are displayed, along with corresponding −log_10_ *p* values and gene numbers. (C) Bar plot of GO analysis for upregulated DEGs between nEB‐NSCs grafted for 6 months and saline‐injected control samples. The top enriched biological processes related to neurogenesis are displayed, along with corresponding fold enrichment values, adjusted *p*‐values, and gene numbers. Fold Enrichment: GeneRatio/BgRatio. (D) Enrichment map showing term‐term network of GO terms derived from upregulated DEGs between nEB‐NSCs and Saline at 6 months post‐transplantation.
**Figure S5:** nEB‐NSCs‐derived factors promote endothelial cell tube formation in vitro. (A, B) Endothelial cells were incubated with endothelial cell medium blankor with nEB‐NSCs‐conditioned medium (scale bar = 500 μm), prepared by collecting medium from nEB‐NSCs and mixing it with endothelial cell medium at a 1:1 ratio; scale bar = 500 μm. (C) Quantitative analysis of endothelial tube formation. nEB‐NSCs enhanced multiple angiogenic metrics, including tube formation rate, vessel area/percentage, total vessel length, endpoints, and junctional density, compared to the blank medium control; *p* < 0.05; *n* = 3 for each group.
**Figure S6:** tMCAO model and evolution of infarct volume. (A) Representative image showing brain regional injury 7 days and 14 days after tMCAO, predominantly involving the cortex and striatum. Red: GFAP, green: NeuN; scale bar = 1000 μm. (B) MRI images and quantification showing continued evolution of infarct volume at 14 days, 1 month, and 3 months after tMCAO, scale bar = 1 mm. (C) Immunostaining and quantification of mouse brain showing that, compared with 7 days, Ly6G‐positive neutrophils decreased 14 days post‐tMCAO. White: CD11b, green: Ly6G; scale bar = 50 μm.

## Data Availability

The data that support the findings of this study are available from the corresponding author upon reasonable request.
