# Supplementary material for: Lineage‐Biased Neural Stem Cell Grafting Promotes Neuronal Differentiation and Vascular Repair in the Chronic Phase of Stroke
Source: CNS Neurosci Ther. 2026 Jan 4;32(1):e70701. doi: 10.1002/cns.70701 (PMC12765984; doi:10.1002/cns.70701)
Supplement: Supplementary file 1 — Table S1: Detailed formulation of NIM and NPM. Table S2: Antibodies used for immunofluorescence and fluorescence‐activated cell sorting (FACS). Table S3: Primer sequences used for RT‐PCR. Table S4: Secondary antibodies used in this study. Figure S1: nEB‐NSCs exhibit higher neuronal differentiation and functional repair potential compared with EB‐NSCs. (A) PCA analysis of ESCs, EB‐NSCs, and nEB‐NSCs (n = 3). (B) Heatmap depicting the relative expression of ESC marker genes. (C) Heatmap showing genes associated with CNS expression among ESCs, EB‐NSCs, and nEB‐NSCs. (D) Volcano plot showing DEGs between EB‐NSCs and nEB‐NSCs. Significantly upregulated and downregulated genes (p < 0.05, |log2FC| > 1) are shown in red and blue, respectively. NSCs marker genes were specifically labeled. (E) Bar plot for GO analysis of upregulated DEGs between EB‐NSCs and nEB‐NSCs. The top enriched biological processes related to the vasculature are displayed, along with corresponding −log10 p values and gene numbers. (F) Volcano plot showing DEGs between EB‐NSCs and nEB‐NSCs. Significantly upregulated and downregulated genes (p < 0.05, |log2FC| > 1) are indicated in red and blue, respectively. Vasculature marker genes are specifically labeled. (G) Bar plot for GO analysis of upregulated DEGs between EB‐NSCs and nEB‐NSCs. The top enriched biological processes related to neurogenesis are displayed, along with corresponding −log10 p values and gene numbers. Figure S2: Transplanted nEB‐NSCs exhibit a neuronal fate and express mature neuronal markers. (A) Representative images 1 month post‐transplantation showing nEB‐NSCs expressing the neural marker PAX6 (green), the human‐specific transplantation marker STEM121 (red), and nuclear counterstain DAPI (blue); scale bar = 20 μm. (B) Representative images 3 months post‐transplantation showing nEB‐NSCs expressing the mature neuronal marker NeuN (green) and STEM121 (red); scale bar = 50 μm. (C) Representative images 6 months post‐transplantation show [file CNS-32-e70701-s001.docx]

**Tables**

**TABLE S1.** Detailed formulation of NIM and NPM.

| **Name** | **Components** | **Amount** |
| --- | --- | --- |
| NIM | DMEM/F12 | 24 mL |
|  | Neurobasal | 24 mL |
|  | N2 supplement | 1 mL |
|  | B27 supplement | 500 μL |
|  | GlutaMAX | 500 μL |
|  | SB431542 | 10 μM |
|  | LDN193189 | 500 nM |
| Total vol. |  | 50 mL |
| NPM | DMEM/F12 | 24.375 mL |
|  | Neurobasal | 24.375 mL |
|  | N2 supplement | 500 μL |
|  | B27 supplement | 250 μL |
|  | GlutaMAX | 500 μL |
| Total vol. |  | 50 mL |

NIM: Neural Induction Medium, a specialized medium designed to promote NSC differentiation; NPM: Neural Preservation Medium, a basal medium used for maintaining NSC viability.

**TABLE S2.** Antibodies used for immunofluorescence and fluorescence-activated cell sorting (FACS).

| **Antibody** | | **Antibody Host** | | **Cat#** | **Supplier** | **Dilution** |
| --- | --- | --- | --- | --- | --- | --- |
| CD31 | | Mouse | | Ab24590 | Abcam | 1:200 |
| ChAT | | Rabbit | | ab114P | Abcam | 1:100 |
| DARPP32 | | Rabbit | | Ab40801 | Abcam | 1:500 |
| FITC -PAX6 | | Human | | 561664 | BD | 1:200 |
| GABA | | Rabbit | | A2052 | Sigma-Aldrich | 1:500 |
| GFAP | Rat | | 13-0300 | | Invitrogen | 1:800 |
| HuNu | | Mouse | | Ab1981 | Abcam | 1:200 |
| MAP2 | | Rabbit | | ab32454 | Abcam | 1:200 |
| NeuN | | Rabbit | | Ab177487 | Abcam | 1:400 |
| Oligo2 | | Rabbit | | Ab9610 | Abcam | 1:200 |
| PE-SOX2 | | Human | | 562195 | BD | 1:200 |
| SOX10 | | Goat | | AF2864 | Biotechno | 1**:**100 |
| STEM121 | | Mouse | | Y40410 | Takara | 1:500 |
| vGLUT1 | | Rabbit | | 482400 | Invitrogen | 1:1000 |

**TABLE S3.** Primer sequences used for RT-PCR.

| **Gene name** | **Forward sequence (5'–3')** | **Reverse sequence (3'–5')** |
| --- | --- | --- |
| hPAX6 | TCCATCAGTTCCAACGGAGAA | GTGGAATTGGTTGGTAGACAC |
| hOLIG2 | GATAGTCGTCGCAGCTTTCG | CCTGAGGCTTTTCGGAGC |
| hSOX10 | CCACCTATGCCACAGTGCCTAAG | GTGCCAACTCCTTCCTGCCTTC |
| hGLAST | AGAACAATGGCGTGGACAAGC | AATGGCAGCCAAAGCCTCAT |
| mAPOLD1 | TGAATCTTGGCCTTCAGCAC | CTACTTCATCGTCTTCTTTGGCT |
| mVEGF | TGGTGACATGGTTAATCGGTC | GGCAGCTTGAGTTAAACGAAC |
| mGAPDH | TCTTGCTCAGTGTCCTTGC | CTTTGTCAAGCTCATTTCCTGG |
| hGAPDH | TGTAGTTGAGGTCAATGAAGGG | ACATCGCTCAGACACCATG |

hPAX6: Human Paired Box 6; hOLIG2: Human Oligodendrocyte Transcription Factor 2; hSOX10: Human SRY-Box Transcription Factor 10; hGLAST: Human Glutamate Aspartate Transporter; mAPOLD1: Mouse Apolipoprotein L Domain Containing 1; mVEGF: Mouse Vascular Endothelial Growth Factor; mGAPDH: Mouse Glyceraldehyde-3-Phosphate Dehydrogenase; hGAPDH: Human Glyceraldehyde-3-Phosphate Dehydrogenase.

**TABLE S4.** Secondary antibodies used in this study.

| **Antibody** | **Cat#** | **Supplier** | **Dilution** |
| --- | --- | --- | --- |
| Goat anti-Mouse IgG (H+L)  Alexa Fluor^TM^ 568 | A-11004 | Invitrogen | 1:2000 |
| Donkey anti-Rabbit IgG (H+L)  Alexa Fluor^TM^ 488 | A-21206 | Invitrogen | 1:2000 |
| Goat anti-Rat IgG (H+L)  Alexa Fluor^TM^ 647 | A-21247 | Invitrogen | 1:2000 |
| Donkey anti-Goat IgG (H+L)  Alexa Fluor^TM^ 647 | A-21447 | Invitrogen | 1:2000 |

**Figures:**


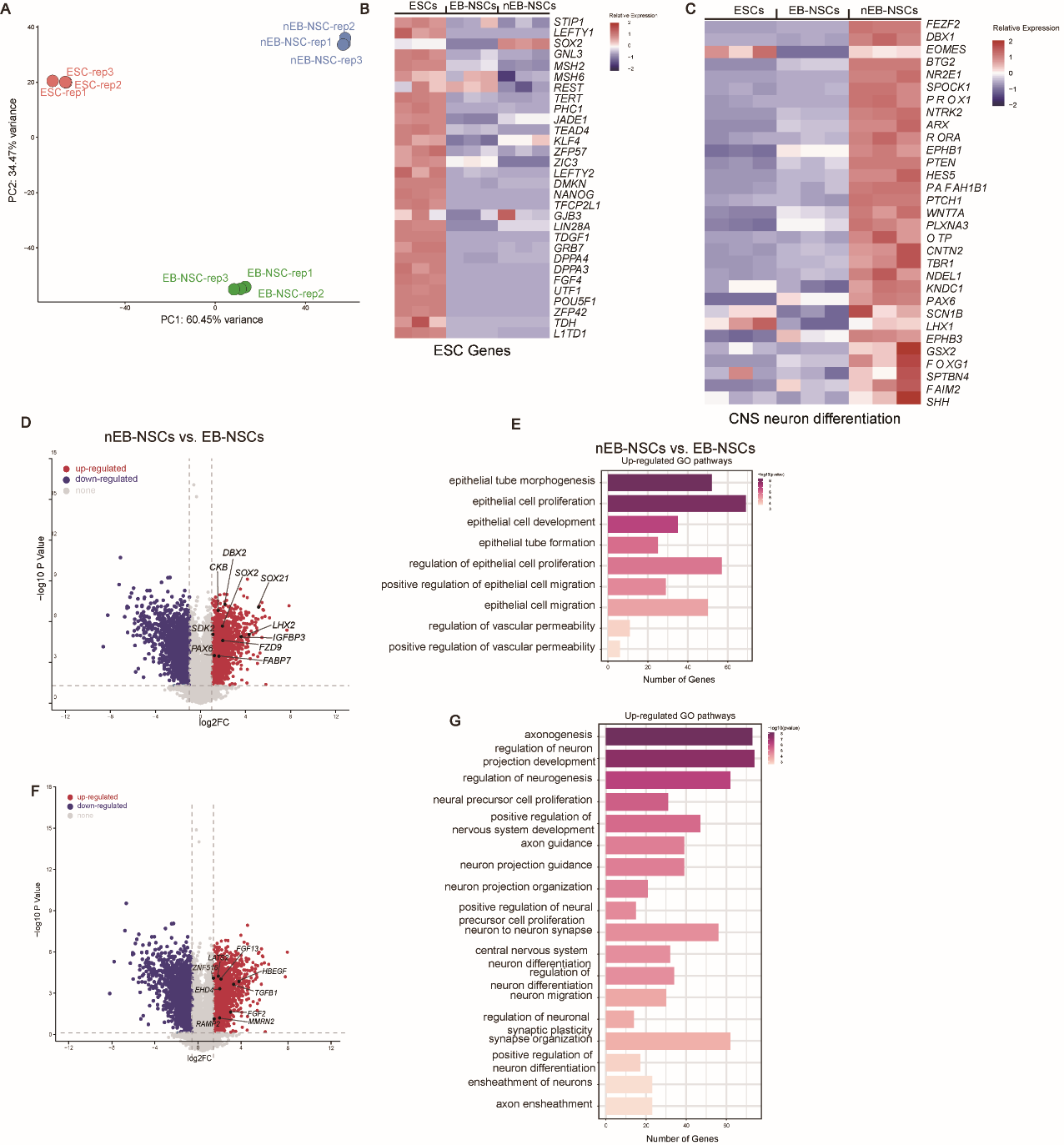


**FIGURE S1.** nEB-NSCs exhibit higher neuronal differentiation and functional repair potential compared with EB-NSCs. (A) PCA analysis of ESCs, EB-NSCs, and nEB-NSCs (n = 3). (B) Heatmap depicting the relative expression of ESC marker genes. (C) Heatmap showing genes associated with CNS expression among ESCs, EB-NSCs, and nEB-NSCs. (D) Volcano plot showing DEGs between EB-NSCs and nEB-NSCs. Significantly upregulated and downregulated genes (p < 0.05, |log2FC| > 1) are shown in red and blue, respectively. NSCs marker genes were specifically labeled. (E) Bar plot for GO analysis of upregulated DEGs between EB-NSCs and nEB-NSCs. The top enriched biological processes related to the vasculature are displayed, along with corresponding -log_10_ p values and gene numbers. (F) Volcano plot showing DEGs between EB-NSCs and nEB-NSCs. Significantly upregulated and downregulated genes (p < 0.05, |log2FC| > 1) are indicated in red and blue, respectively. Vasculature marker genes are specifically labeled. (G) Bar plot for GO analysis of upregulated DEGs between EB-NSCs and nEB-NSCs. The top enriched biological processes related to neurogenesis are displayed, along with corresponding -log10 p values and gene numbers.


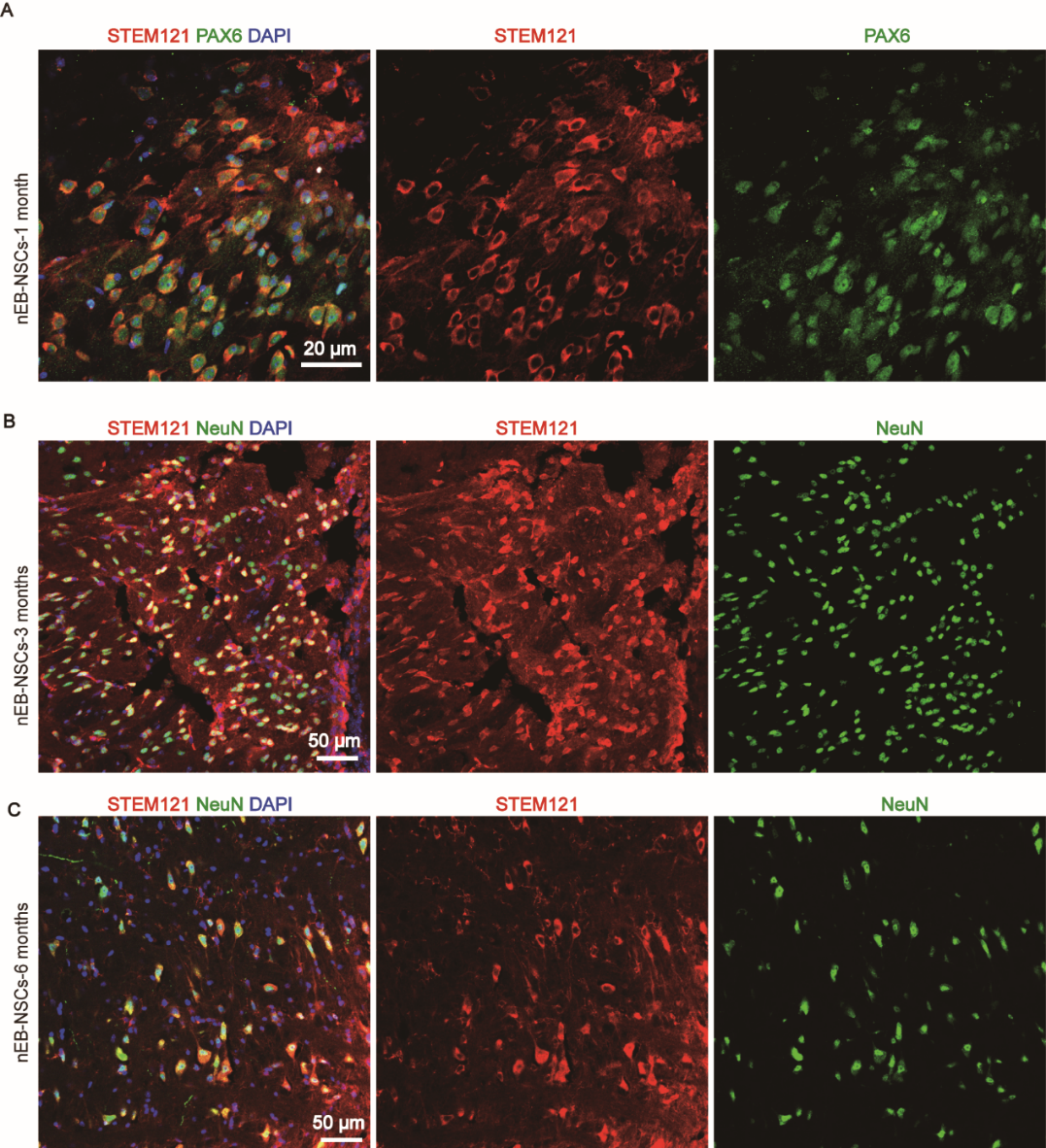


**FIGURE S2.** Transplanted nEB-NSCs exhibit a neuronal fate and express mature neuronal markers. (A) Representative images 1 month post-transplantation showing nEB-NSCs expressing the neural marker PAX6 (green), the human-specific transplantation marker STEM121 (red), and nuclear counterstain DAPI (blue); scale bar = 20 μm. (B) Representative images 3 months post-transplantation showing nEB-NSCs expressing the mature neuronal marker NeuN (green) and STEM121 (red); scale bar = 50 μm. (C) Representative images 6 months post-transplantation showing nEB-NSCs expressing the mature neuronal marker NeuN (green) and STEM121 (red); scale bar = 50 μm.


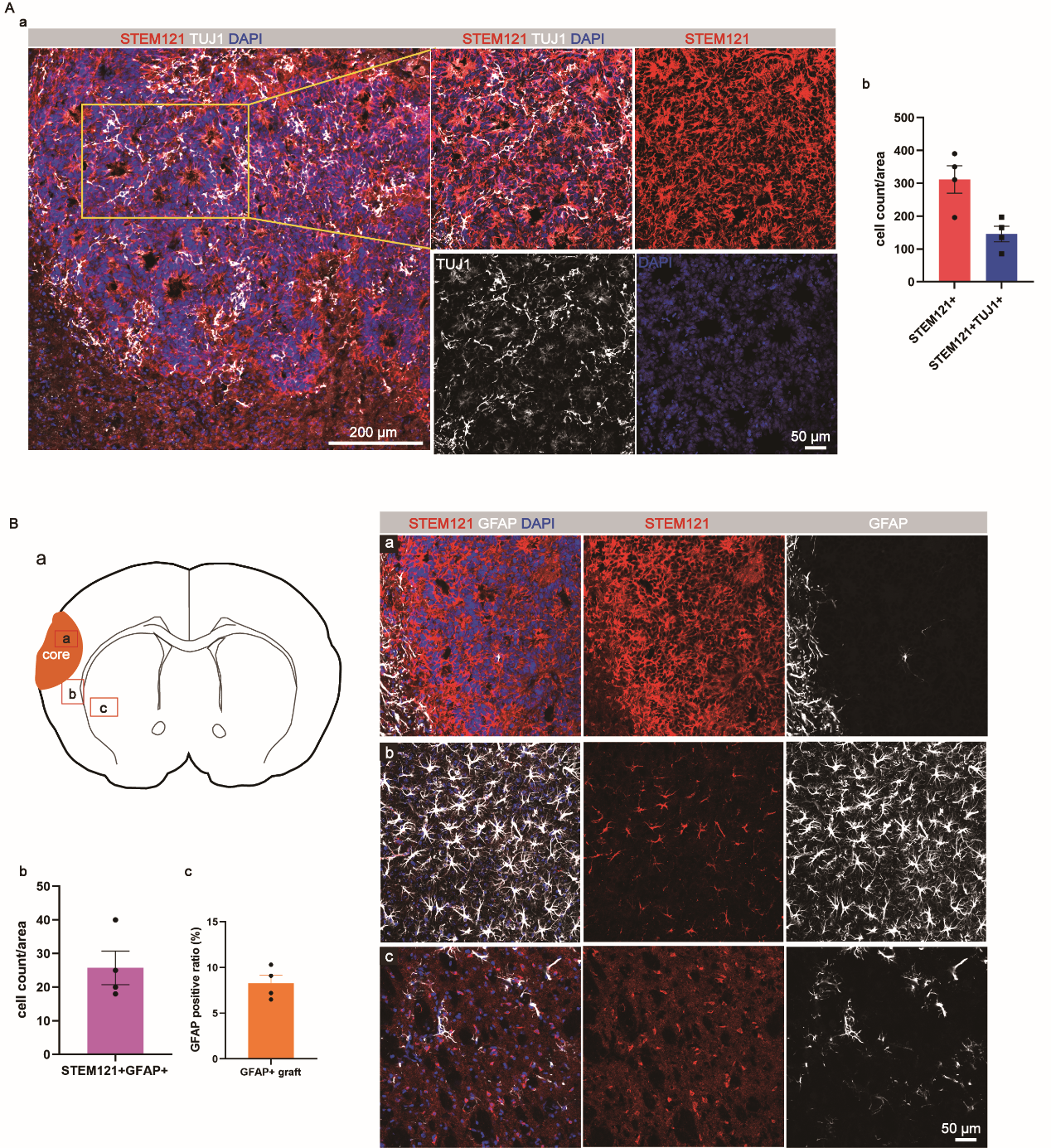


**FIGURE S3.** EB-NSCs transplanted into ischemic stroke mice differentiate into both neuronal and astrocytic lineages. (A) Immunostaining and quantification of EB-NSCs expressing the immature neuronal marker TUJ1 and the human-specific transplantation marker STEM121. Nuclei are stained with DAPI. (B) Immunostaining and quantification of EB-NSCs expressing the astrocyte marker GFAP (white) and STEM121 (red). Nuclei are stained with DAPI. Brain slices were collected at 7 days post-transplantation: n = 4.

**
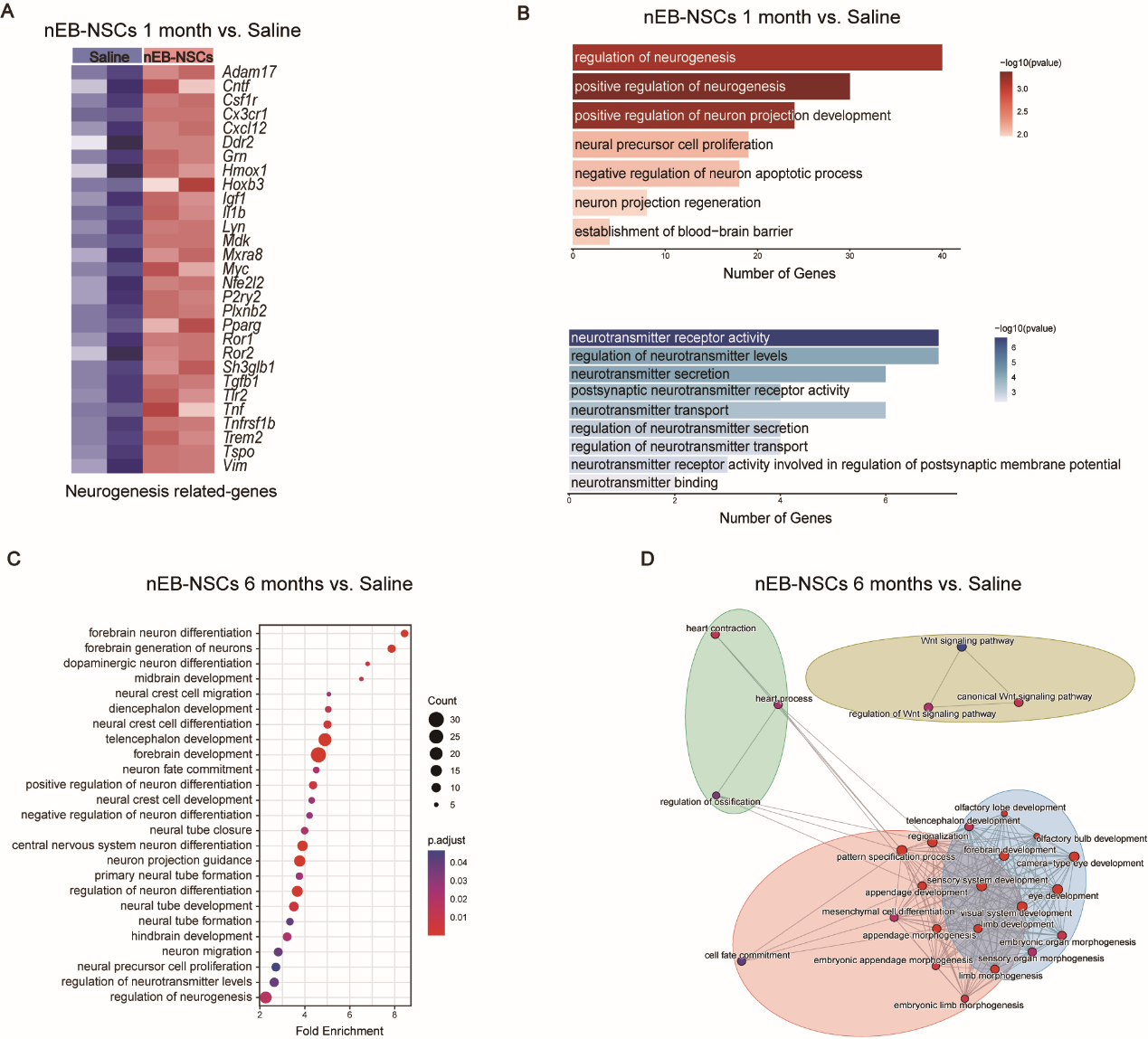
**

**FIGURE S4.** nEB-NSCs transplantation facilitates nerve regeneration in the ischemic brain. (A) Heatmap depicting the relative expression of neurogenesis-related genes between nEB-NSCs grafted for 1 month and saline-injected control samples. (B) Bar plot for GO analysis of upregulated DEGs (top panel) and downregulated DEGs (bottom panel) between nEB-NSCs grafted for 1 month and saline-injected control samples. The top enriched biological processes related to neurogenesis are displayed, along with corresponding -log10 p values and gene numbers. (C) Bar plot of GO analysis for upregulated DEGs between nEB-NSCs grafted for 6 months and saline-injected control samples. The top enriched biological processes related to neurogenesis are displayed, along with corresponding fold enrichment values, adjusted p-values, and gene numbers. Fold Enrichment: GeneRatio/BgRatio. (D) Enrichment map showing term-term network of GO terms derived from upregulated DEGs between nEB-NSCs and Saline at 6 months post-transplantation.


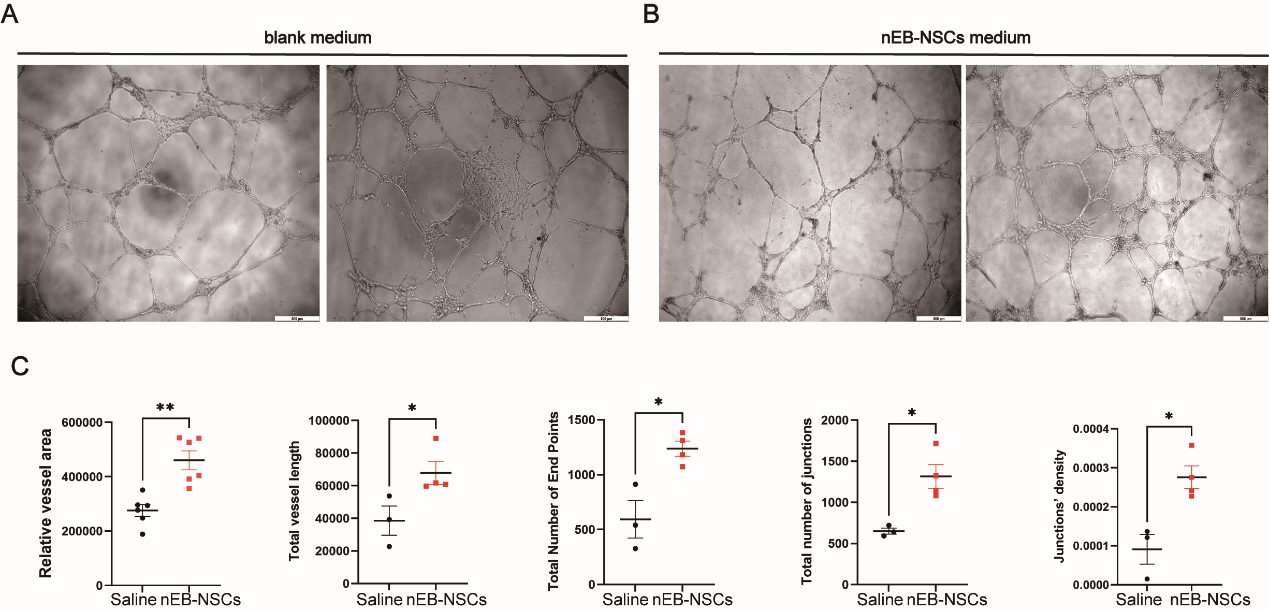


**FIGURE S5.** nEB-NSCs-derived factors promote endothelial cell tube formation in vitro. (A, B) Endothelial cells were incubated with endothelial cell medium blankor with nEB-NSCs-conditioned medium (scale bar = 500 µm) , prepared by collecting medium from nEB-NSCs and mixing it with endothelial cell medium at a 1:1 ratio; scale bar = 500 µm. (C) Quantitative analysis of endothelial tube formation. nEB-NSCs enhanced multiple angiogenic metrics, including tube formation rate, vessel area/percentage, total vessel length, endpoints, and junctional density, compared to the blank medium control; p < 0.05; n = 3 for each group.


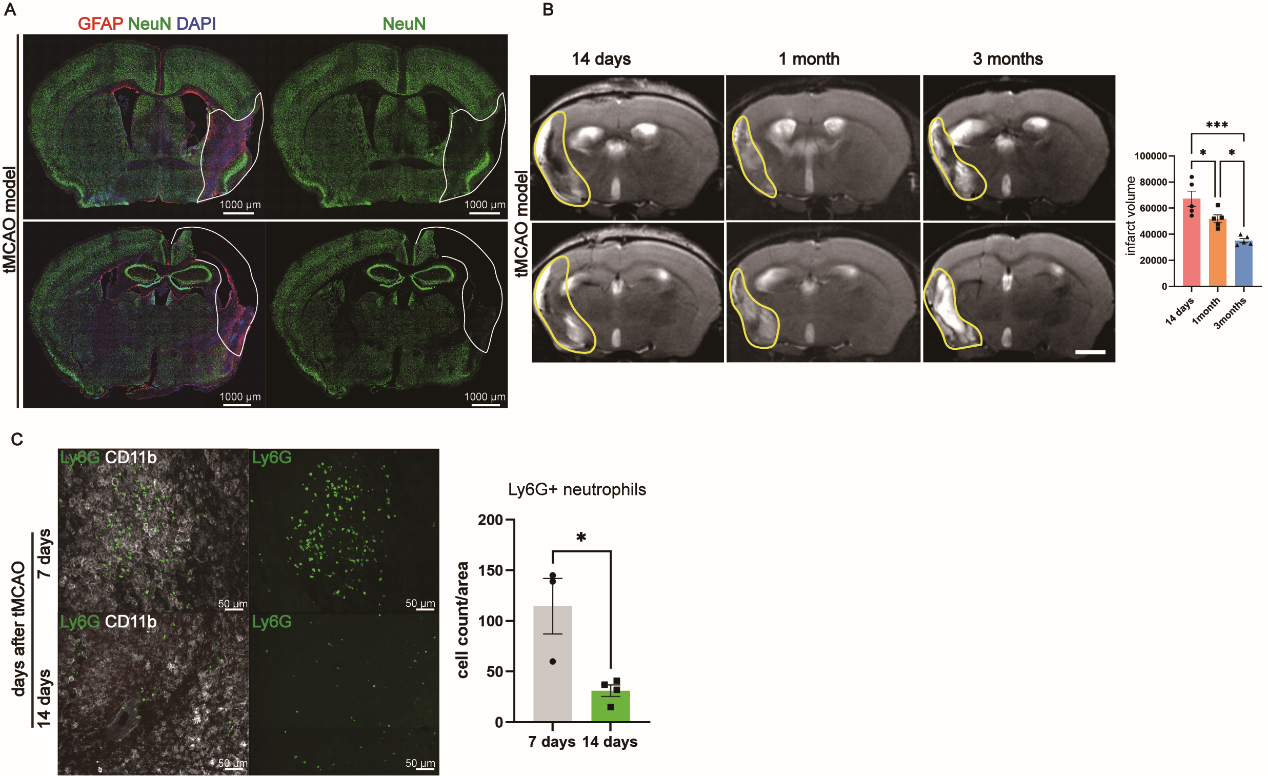


**FIGURE S6**. tMCAO model and evolution of infarct volume. (A) Representative image showing brain regional injury 7 days and 14 days after tMCAO, predominantly involving the cortex and striatum. Red: GFAP, green: NeuN; scale bar = 1000 µm. (B) MRI images and quantification showing continued evolution of infarct volume at 14 days, 1 month, and 3 months after tMCAO, scale bar = 1mm. (C) Immunostaining and quantification of mouse brain showing that, compared with 7 days, Ly6G-positive neutrophils decreased 14 days post-tMCAO. White: CD11b, green: Ly6G; scale bar = 50 µm.
